# Associations of Cognitive Fusion and Pain Catastrophizing with Fibromyalgia Impact through Fatigue, Pain Severity, and Depression: An Exploratory Study Using Structural Equation Modeling

**DOI:** 10.3390/jcm9061763

**Published:** 2020-06-06

**Authors:** Carmen Écija, Octavio Luque-Reca, Carlos Suso-Ribera, Patricia Catala, Cecilia Peñacoba

**Affiliations:** 1 Department of Psychology, Rey Juan Carlos University, 28922 Alcorcón, Madrid, Spain; carmen.ecija@urjc.es (C.E.); octavio.luque@urjc.es (O.L.-R.); patricia.catala@urjc.es (P.C.); 2 Department of Basic and Clinical Psychology and Psychobiology, Jaume I University, 12071 Castelló de la Plana, Castelló, Spain; susor@uji.es

**Keywords:** fibromyalgia syndrome, cognitive factors, physical symptoms, chronic pain, depressive symptoms, function limitations, structural equation modeling

## Abstract

Differences in fibromyalgia impact on functioning exist and appear to be influenced by numerous factors, including symptomatology severity, as well as the cognitive profile of the individual. The contribution of these elements, however, tends to be explored in a fragmented manner. To address this issue, we tested a comprehensive structural equation model in which associations of cognitive fusion and pain catastrophizing with function limitations are investigated through fibromyalgia symptomatology (i.e., fatigue, pain severity, and depression) in 231 women with fibromyalgia. In the model, cognitive fusion and two catastrophizing components (magnification and helplessness) were associated with poorer functioning indirectly through fibromyalgia symptomatology. Only the rumination component of catastrophizing had a direct association with functional limitations. All fibromyalgia symptoms were linked to increased functional limitations. A parsimonious model with significant associations only obtained an excellent fit (S-B *χ*^2^ = 774.191, df = 543, *p* < 0.001; CFI = 0.943; RMSEA = 0.043; CAIC = −2724.04) and accounted for 50% of the variance of functional limitations. These results suggest that the relationship between psychological cognitive processes, fibromyalgia symptomatology, and functional limitations is complex and support the need for comprehensive models such as the present. The findings are discussed in the context of personalized psychological treatments (i.e., the need to address certain cognitive processes according to the problematic symptomatology or outcome).

## 1. Introduction

Functioning is significantly impaired in people with fibromyalgia (FM). It is widely accepted that a moderate-to-strong association exists between functional limitation and pain severity [1,2,3]. However, pain severity is not the only factor associated with the impact of the syndrome and functional limitations are known to vary across patients reporting similar pain severity levels [4,5]. Thus, it has been argued that functional limitation in patients with FM and other pain conditions should be conceptualized within a more comprehensive biopsychosocial perspective [6]. In this line, fatigue and depression have been repeatedly associated with poorer functioning in people with FM [7,8], which explains why both were added as part of the newer diagnostic criteria published in 2010 by the American College of Rheumatology [9].

While acknowledging the important role of FM symptomatology (e.g., pain severity, fatigue, and depression) in daily functioning, in the past years, researchers have been encouraged to investigate the mechanisms that explain individual differences in this symptomatology and, ultimately, in overall functioning (i.e., mediators), as these would be the actual targets during interventions [10]. In this sense, several models of pain have pointed to cognitive factors as key elements explaining individual differences in symptomatology and functionality in chronic pain populations, such as FM [11]. For example, according to the fear-avoidance model of pain, aggravation of symptomatology and disability in the presence of pain occurs when individuals catastrophize about their pain experience [12]. Specifically, because catastrophizers tend to magnify the threat associated with pain; feel helpless in the face of pain; and show higher pain levels, poorer functionality, and increased mental distress [13].

Another well-established model of pain that has contributed to the understanding of functioning in people with chronic pain is the psychological flexibility model. Different to the fear-avoidance model, the psychological flexibility model of pain shifts the emphasis from thought-content restructuring to changing the functional relationship with thoughts without challenging their content [14]. In this model, the focus is shifted from pain catastrophizing (a thought content) to cognitive fusion, which is conceptualized as the tendency to being trapped by the meaning ascribed to internal events, such as thoughts, emotions, and images (functional relationship with thoughts, irrespective of their content). In this sense, the psychological flexibility model suggests that cognitive fusion would explain the inability of some individuals to implement adaptive coping methods in the presence of pain or related symptomatology [15]. Indeed, there is evidence to support the influence of cognitive fusion on outcomes in patients with FM [16,17]. Research supports the idea that both processes are distinct and that they could potentially interact and influence each other [18].

In sum, the reviewed literature suggests that FM symptomatology (e.g., pain, fatigue, and depression) is closely interrelated and associated with functionality. Additionally, individual differences in symptomatology and functionality are influenced by other biopsychosocial components, such as cognitive factors. Several models of pain have been proposed to explain such complex associations between variables. However, to the best of our knowledge a comprehensive model that includes both the fear-avoidance and the psychological flexibility model and the combination of symptoms and functioning in a structural equation model is missing. This is important to investigate the complex associations between involved factors hypothesized in the literature.

**Figure 1:**
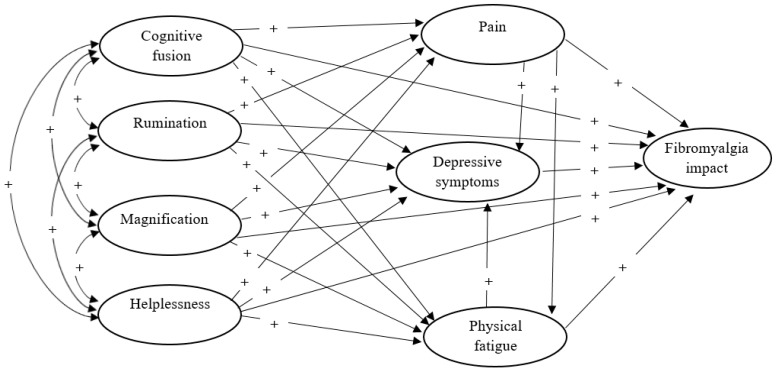
Hypothesized model of cognitive fusion and pain catastrophizing associations with fibromyalgia impact through physical fatigue, pain severity, and depression. The “+” symbol represents an expected positive association.

In the light of the previous literature [1,7,8,13,17], our goals is to test the hypothesized associations of pain catastrophizing, cognitive fusion, and FM symptom severity (pain, fatigue, and depression) in a theoretically-sound model that accounts for the contribution of these elements altogether in relation to functional limitation. We hypothesize that catastrophizing components and cognitive fusion will be associated with greater disability (see Figure 1). We also anticipate that pain, fatigue, and depression will mediate the aforementioned relationship between cognitive factors and functional limitations. Because we expect that study variables will be intercorrelated and will share common variance in relation to functional limitation, we anticipate that only a reduced number of independent variables will be uniquely associated with functional limitation when all variables are considered altogether in the same model. Because such complex models remain unexplored in this population, anticipating which paths will remain significant is difficult at this stage, and this will be investigated in an exploratory manner. A more parsimonious model will be proposed as a post hoc to the exploratory analyses.

## 2. Methods

### 2.1. Participants

The sample comprised 231 women with FM (mean age = 56.91 years, SD = 8.9 years, range 30–78 years). Their sociodemographic characteristics are shown in Table 1. The eligibility criteria to participate in the present study included having a diagnosis of FM according to the American College of Rheumatology (ACR) criteria [9,19], being over 18 years of age, and providing a written consent to participate in the investigation. Patients were recruited from different associations in Spain (Madrid, Ciudad Real, Albacete, Guadalajara, and Toledo).

The study followed the ethical principles for research with human participants (Helsinki Declaration) and was approved by the University Ethics Committee (Ref. PI17/00858).

### 2.2. Measures

#### 2.2.1. Pain Catastrophizing

The Spanish version of the Pain Catastrophizing Scale was used to evaluate the three components of pain catastrophizing, understood as an exaggerated negative orientation towards actual or anticipated pain experiences, namely rumination, magnification, and helplessness [20,21]. Rumination includes ruminative thoughts, worry, and an inability to inhibit pain-related thoughts; magnification relates to an exaggeration of the unpleasantness of pain situations and expectancies for negative outcomes; and helplessness refers to negative appraisals related to the ability to deal effectively with painful stimuli. This 13-item scale with a 4-point Likert response format contains items such as “I keep thinking about how badly I want the pain to stop”, “I become afraid that the pain may get worse”, or “there is nothing I can do to reduce the intensity of the pain”, for rumination, magnification and helplessness, respectively. Higher scores on the scales represent higher catastrophizing. In the present study, the internal consistency of the three scales was adequate (0.88, 0.78, and 0.89 for rumination, magnification, and helplessness, respectively).

#### 2.2.2. Cognitive Fusion

The Spanish version of the Cognitive Fusion Questionnaire was used to assess cognitive fusion, which is conceived as the degree to which a person is psychologically tangled with and dominated by the form or content of its thoughts [22,23]. High scores in this 7-item scale with a 7-point Likert response format indicate high cognitive fusion. The scale contains items like “I get so caught up in my thoughts that I am unable to do the things that I most want to do”. The internal consistency obtained in this study was excellent (0.91).

#### 2.2.3. Pain Severity

To assess pain severity, we used the mean score of the four pain severity items from the Brief Pain Inventory [24]: maximum, minimum, and overall pain intensity during the last 7 days and pain intensity at the current time. Each rating is evaluated using an 11-point numerical scale (0 = “no pain” and 10 = “the worst pain you can imagine”). This procedure to measure pain severity has been widely used in the pain literature [25]. In this study, the internal consistency of this scale was high (0.86).

#### 2.2.4. Depressive Symptoms

The Depression subscale of the Spanish version of the Hospital Anxiety and Depression Scale (HADS) was used to evaluate the severity and presence of depressive symptoms over the preceding week [26]. The HADS is a brief and widely used instrument to measure the possible presence of anxiety and depressive states in medical, non-psychiatric outpatient clinic settings. High scores in this 7-item subscale with a 4-point Likert response format denote high depressive symptomatology. The scale is composed of items such as “I have lost interest in my appearance”. The internal consistency of this subscale was high (0.85) in the present study.

#### 2.2.5. Physical Fatigue

The Physical Fatigue dimension of the Spanish version of the Multidimensional Fatigue Inventory (MFI) was used [27]. This questionnaire is a 20-item assessment tool with a 5-point Likert response format that evaluates five domains of fatigue (general fatigue, physical fatigue, mental fatigue, reduced motivation, and reduced activity). High scores in this 4-item dimension indicate a high degree of fatigue symptoms that are physical in nature. This subscale contains items like “physically, I feel only able to do a little”. The selection of this dimension was motivated by its conceptualization of fatigue as a physical symptom, in agreement with the proposed theoretical model and consistent with previous literature on the relationship between fatigue and functionality [28]. Although the rest of the MFI dimensions contribute to assess fatigue in all its complexity, they were discarded because they reflect slightly different (although related) conceptualizations from those intended in this work, such as cognitive (mental fatigue dimension) or affective-emotional (reduced motivation dimension) aspects. Furthermore, the conceptual overlap of both the reduced activity dimension with functionality measures [29] and the general fatigue dimension with pain measures [30] led to discard their use to avoid overlaps with other variables used in the study. In the present work, the internal consistency of this subscale was good (0.73).

#### 2.2.6. FM Impact

We used the “overall impact” dimension of the Spanish adaptation of the Revised Fibromyalgia Impact Questionnaire (FIQ-R) to evaluate the impact of FM on functioning [31,32]. In the FIQ-R, the two items that compose the “overall impact” dimension are answered on an 11-point numerical rating scale from 0 to 10, with different verbal anchors depending on the item. Higher scores represent higher impact perception. Note that this dimension has two items only (“fibromyalgia prevented me from accomplishing goals for the week” and “I was completely overwhelmed by my fibromyalgia symptoms”) and three are usually recommended for scale development. However, the use of reduced, even single-item measures is frequent and has shown to be psychometrically valid in the pain literature [33]. This was the case of the “overall impact” scale in the present study, which obtained a high internal consistency score (0.81).

### 2.3. Statistical Analyses

The Statistical Package for Social Sciences IBM SPSS for Windows, version 22.0 (Armonk, NY, USA) [34] was used for descriptive analyses, and EQS for Windows, version 6.2 (Encino, CA, USA) [35] was used to conduct structural equation modeling analyses.

Preliminarily, both an analysis of missing values and an evaluation of data normality were performed. The variables of interest contained some missing values (range 0.4% to 1.7%) that did not exceed 5% of the initial sample (*n* = 234), so data missingness did not compromise the reliability of scores [36]. As suggested by the Little’s test (*χ*^2^ = 274.01, df = 257, *p* = 0.22), missing data appeared to be missing completely at random (MCAR). Because all missing data provided from 3 participants only, we eliminated them from the analyses (listwise deletion), as recommended in the literature [37]. This resulted in a final sample size of 231 patients.

Convergent validity and reliability of the variables included in the structural equation model were determined assessing the average variance extracted (AVE) and composite reliability (CR), respectively. AVE over 0.50 and CR scores over 0.70 are considered good [38], while AVE values slightly below 0.50 are also acceptable as long as CR is over 0.60 [39]. An alpha level of 0.05 was selected in the present study despite the number of tests conducted due to the exploratory nature of the analyses. Using more restrictive alpha levels, for example by implementing a Bonferroni–Holm correction, is less advisable in exploratory studies as they increase the risk of false negatives by attempting to reduce the risk of false positives [40]. The present study findings should serve guide future confirmatory models in which such corrections would be more sensible.

A model development strategy was followed in this study, using the robust maximum likelihood estimation method. The goodness of fit of the model was assessed by: (1) the Satorra–Bentler scaled *χ*^2^ statistic (S-B *χ*^2^), its degrees of freedom (df), and *p* values; (2) the Comparative Fix Index (CFI), as an incremental fit index; (3) the Root Mean Square Error of Approximation (RMSEA) with its 90% confidence interval (CI); and (4) the Consistent Akaike Information Criterion (CAIC), as a parsimony index to compare non-nested models. Considering the sample size and the number of observed variables, adequate model fit was determined using the following cutoffs: CFI > 0.92, RMSEA < 0.08, and CAIC of less magnitude indicating greater parsimony [37,38]. Regarding S-B *χ*^2^, although non-significant *p* values indicate good fit, with complex models such as the current one, a model can be considered adequate if the rest of the fit indices reach the cut-off values [37]. Finally, the Harman’s single-factor test [41] was employed in order to test whether common method variance was an issue.

## 3. Results

This was a cross-sectional survey performed in people with FM belonging to patients’ associations. Of them, 268 agreed to participate in the study and met our inclusion criteria. Finally, a total of 231 patients constituted the final sample (25 did not attend the scheduled assessment appointment, nine questionnaires were left blank, and 3 questionnaires contained missing data that could not be retrieved because participants could no longer be reached).

Regarding the measurement model, all items were included as observed variables. However, two of them (one on the depressive symptomatology scale and another on the physical fatigue scale) yielded reduced factor loadings and were eliminated. The measurement model consisted of 35 observed variables grouped into eight latent variables or latent factors. In all cases, we obtained adequate scores both in the factor loadings of the observed variables and in the AVE and the CR of the factors (see Table 2). Finally, the model showed good fit (S-B *χ*^2^ = 764.331, df = 532, *p* < 0.001; CFI = 0.942; RMSEA = 0.044 (90% CI from 0.036 to 0.050)).

**Figure 2:**
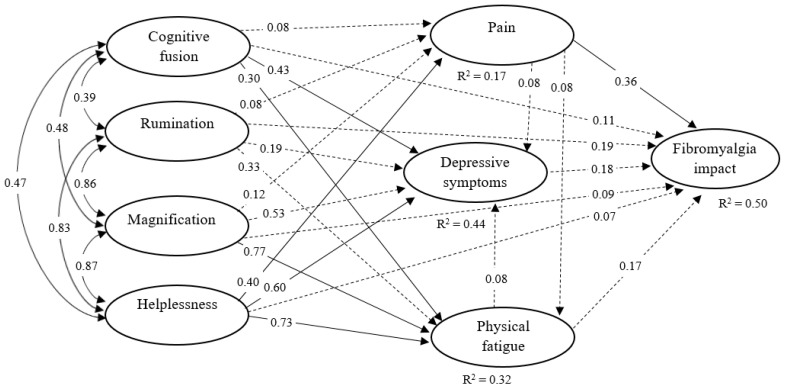
Standardized coefficients of the hypothesized model (M1). Dashed lines represent non-significant paths. The latent factors of the model are shown, while the observed indicators and measurement errors have been omitted for space and clarity reasons. The squared multiple regression coefficients (R^2^) reflect the amount of factor variance associated with variance of its independent variables.

With regard to the structural model, the hypothesized model (M1) obtained an adequate fit (S-B *χ*^2^ = 772.023, df = 533, *p* < 0.001; CFI = 0.940; RMSEA = 0.044 (90% CI from 0.037 to 0.051); CAIC = −2661.786) and accounted for 50% of the impact of FM. However, this first model could not confirm many of the initially hypothesized relationships between variables (see Figure 2 and Table 3). Specifically, the non-significant pathways were from rumination and magnification to depressive symptoms, pain severity, and FM impact; from rumination to physical fatigue; from helplessness to FM impact; from cognitive fusion to both pain and FM impact; from pain severity to both depressive symptoms and physical fatigue; from depressive symptoms to FM impact; and from physical fatigue to depressive symptoms and FM impact. While the implications of these findings will be discussed in more detail in the next section, the fact that hypothesized contributions were not confirmed is likely to be explained by the multivariate approach used in the present study, in which the independent variables had to compete between them to account for unique variance of outcomes.

**Figure 3:**
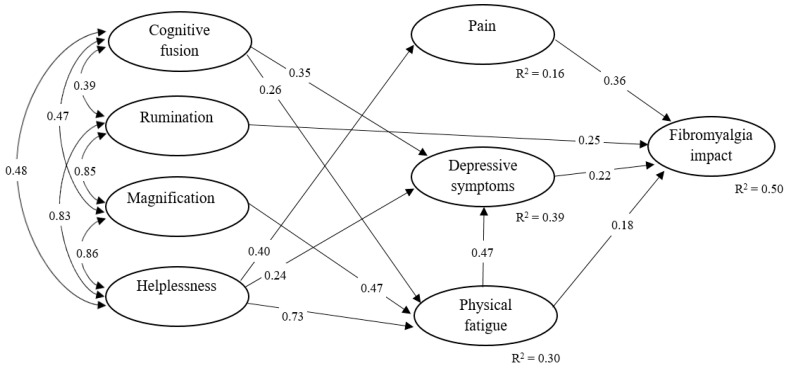
Standardized coefficients of the re-specified model (M2). Only significant paths and the latent factors of the model are shown; the observed indicators and measurement errors have been omitted for space and clarity reasons. The squared multiple regression coefficients (R^2^) reflect the amount of factor variance associated with variance of its independent variables.

The hypothesized model was thus re-specified removing the non-significant pathways to test the adequacy of this modified and arguably more parsimonious model (M2). Figure 3 depicts that this second model achieved a good fit (S-B *χ*^2^ = 774.191, df = 543, *p* < 0.001; CFI = 0.943; RMSEA = 0.043 (90% CI from 0.036 to 0.050); CAIC = −2724.04). Different to the first model, all the relationships in this second model were significant (see Table 3), and the same amount of variance of FM impact was accounted for by the independent variables (i.e., 50%). After analyzing the modification indices, the results did not suggest changing any of the relationships in this re-specified model. Thus, the results of comparing the fit indices and the parsimony index of both structural models suggested the preference for the second model M2.

To conclude, the results provided by the Harman’s single-factor test (S-B *χ*^2^ = 2289.813, df = 560, *p* < 0.001; CFI = 0.571; RMSEA = 0.116 (90% CI from 0.111 to 0.121)) revealed that a single factor could not account for the variance in the present data, implying that common method variance bias was not a critical problem in this study.

## 4. Discussion

Popular models of pain, such as the fear-avoidance and the psychological model of pain, have hypothesized that functional limitations in patients with chronic pain are influenced by a complex interplay between symptomatology (e.g., pain severity, fatigue, and depression) and psychological mechanisms (e.g., catastrophizing and cognitive fusion). Research, however, tends to explore the relationship between the aforementioned variables in a fragmented way (e.g., by including a reduced number of symptoms or psychological mechanisms only or by exploring their relationship with one outcome at the same time without accounting for complex direct and indirect associations). The present study contributes to the existing literature by proposing a more comprehensive model of functional limitations in FM patients that includes a set of important symptoms and psychological mechanisms from different psychological perspectives. Overall, our model was satisfactory in terms of explained variance (50%). Another important finding was that a large number of significant bivariate associations became non-significant when explored in a complex multivariate model. This supports the utility of such complex models that account for shared variance.

Regarding the relationship between FM symptoms and functioning, our structural equations model evidenced an association of symptoms (pain severity, fatigue, and depression) with functional limitation, supporting the extensive research in this regard [42,43]. New to the literature is that all symptoms were uniquely and independently associated with functional limitations in the structural equations model after controlling for the role of the other symptoms and the psychological mechanisms (i.e., catastrophizing and fusion). This is important as it suggests that all these symptoms should be considered in a comprehensive evaluation of FM functionality, which is consistent with their inclusion in the 2010 diagnostic criteria of the American College of Rheumatology [9]. These results are also consistent with past research using an integrative model similar to the present one, in which both physical fatigue and depression symptoms were associated with the quality of life of patients with FM. Thus, even though the study outcomes are different, these results support the idea that the relationship between fibromyalgia symptoms and outcomes is relatively robust [44].

Additionally, our findings evidenced that, while important psychological factors like catastrophizing and cognitive fusion might indeed impact on functioning, symptoms might as well uniquely contribute to functioning even after controlling for these cognitive factors. Indeed and as noted in past research, pain and other symptoms appear to have a relatively inescapable limiting nature when there are very intense, even in the presence of adaptive psychological thinking [45]. While acknowledging the important role of symptom severity on functioning, our model also showed that pain catastrophizing and cognitive fusion might be important factors uniquely associated both with FM symptomatology and functional limitation, thus supporting a vast amount of literature in this regard [46,47]. Interestingly, the contribution of catastrophizing components and cognitive fusion on FM impact on functioning was mostly indirect through FM symptomatology, and each component showed a particular association with each symptom. Specifically, what our model suggests is that the only catastrophizing component that is directly associated with FM impact is rumination. Conversely, magnification would be indirectly associated with FM impact via increased physical fatigue, while helplessness would be indirectly related to FM impact through pain severity, depressive symptoms, and physical fatigue. As noted in past research, “whether these components of catastrophizing work interactively to explain behavior has not been examined in a systematic fashion, but certainly would represent an exciting avenue for future research” [43].

The present study has some limitations. First, the data is based on a cross-sectional design, so it is not possible to establish cause-and-effect inferences. Second, while FM patients were recruited from different associations in Spain, the sample is not representative of the whole population of FM patients in this country. Moreover, we did not include males because of the very small prevalence of this disease in this population, so the results might not be generalizable to males. Similarly, the model was tested with patients reporting very different pain durations. Therefore, the extent to which the relationship between factors differs as a function of pain duration or any other covariate (i.e., moderators) is unclear. Additionally, while we included important psychological mechanisms in pain research, the list is not complete. We expect that the present study will inspire future similar investigations including these and other important psychological processes, such as neuroticism, self-efficacy, coping, and acceptance, to name some examples. Note that future investigations should have a more confirmatory nature if attempting to replicate the present study findings, so paths should be constrained to the ones indicated in the present investigation, and alpha levels should be corrected for multiple testing. Another aspect that could be considered as a limitation refers to the utilization of the outcome variable “overall impact” as opposed to the total FIQ-R score. Note that the FIQ-R is composed by three dimensions, namely, overall functioning, a physical functioning scale that mainly focuses on household chorus, and a measure of symptom severity that includes pain severity and depression. The preference for the “overall impact” dimension was motivated by the study focus on overall functioning as opposed to functioning in specific situations (e.g., household chorus). Additionally, as indicated in past similar research [48], the inclusion of the “symptom severity” scale would have contaminated the relationship between at least two independent variables (i.e., pain severity and depression) and the dependent variable. As a final remark, it is important to note that all measures were obtained through self-report, which means that perceived functioning might differ from objectively-measured functioning (e.g., with accelerometers or performance-based physical fitness tests) [44,49]. Similar to the majority of existing literature, our focus in the present study was on subjective functioning and symptomatology only due to the inherently important nature of the subjective experience on outcomes [50]. However, the debate on whether objective or subjective measures of pain and related outcomes should be used in people with chronic pain has been a matter of concern for decades and multimodal measurement (i.e., a combination of both) appears to be preferable when possible [51].

While acknowledging the aforementioned shortcomings, the present study results might as well have a number of clinical implications. In particular, our findings support the idea that psychological cognitive processes (i.e., catastrophizing and cognitive fusion) are associated with poor outcomes in FM patients, which justifies their inclusion in interdisciplinary treatment programs for FM [52]. Similarly, the results indicated that FM symptomatology (e.g., pain severity, fatigue, and depression) significantly and uniquely contributes to functional limitations, which is again consistent with the literature [42,43]. A novel contribution of the present study was the combination of all these elements in a single structural model to explore the unique association between study variables when accounting for the shared variance between independent variables. This is important as it might guide interventions in a more effective manner (i.e., personalization) [53]. For example, according to our findings, in the presence of a patient with FM with impaired functioning because of increased depression, helplessness and cognitive fusion (due to their associations with depressed mood) and rumination (because of its direct contribution on FM impact) might be potentially important treatment targets. Alternatively, if an individual with FM is largely impaired due to severe pain levels, our results support the idea that a treatment focus on reducing helplessness (because of its unique association with pain severity) and rumination (again due to direct relationship with FM impact) might be appropriate.

## Figures and Tables

**Table 1:** Sociodemographic characteristics of the participants.

Age (30–78 years), mean (*SD*)	56.88	(8.9)
Education level, *n* (%)		
Unfinished studies	32	(13.9)
Primary	123	(53.2)
Secondary (and vocational)	61	(26.4)
University	15	(6.5)
Working status, *n* (%)		
Working	28	(12.1)
Retired not due to pain problems	33	(14.3)
Retired not due to pain problems	41	(17.7)
Sick leave	23	(10.0)
House-working	78	(33.8)
Unemployed	28	(12.1)
Marital status, *n* (%)		
Married or living as a couple	183	(79.2)
Single	12	(5.2)
Separated or divorced	20	(8.7)
Widowed	16	(6.9)
Years (1-46) since fibromyalgia diagnosis, mean (*SD*)	12.17	(12.38)

SD, Standard Deviation.

**Table 2:** Validity, reliability, and estimates of the measurement model.

Measurement Model							
Observed Variable	Factor	AVE	CR	R^2^	B	SE	*β*
Rumination 1	Rumination	0.64	0.88	0.47	0.75	0.07	0.68
Rumination 2				0.78	1.10	0.05	0.89
Rumination 3				0.68	1.00	0.06	0.83
Rumination 4				0.63	0.96	0.06	0.79
Magnification 1	Magnification	0.54	0.78	0.57	0.82	0.06	0.76
Magnification 2				0.59	0.97	0.06	0.77
Magnification 3				0.47	0.94	0.07	0.69
Helplessness 1	Helplessness	0.59	0.89	0.37	0.68	0.06	0.60
Helplessness 2				0.57	0.79	0.06	0.75
Helplessness 3				0.74	1.06	0.05	0.86
Helplessness 4				0.73	1.01	0.05	0.85
Helplessness 5				0.70	0.99	0.06	0.83
Helplessness 6				0.43	0.82	0.07	0.65
Cognitive fusion 1	Cognitive fusion	0.59	0.91	0.49	1.06	0.10	0.70
Cognitive fusion 2				0.51	1.27	0.09	0.71
Cognitive fusion 3				0.48	1.21	0.10	0.69
Cognitive fusion 4				0.54	1.12	0.10	0.74
Cognitive fusion 5				0.61	1.39	0.10	0.78
Cognitive fusion 6				0.76	1.52	0.10	0.87
Cognitive fusion 7				0.74	1.58	0.08	0.89
Physical fatigue 1	Physical fatigue	0.48	0.73	0.41	0.71	0.09	0.64
Physical fatigue 2				0.39	0.69	0.08	0.60
Physical fatigue 3				0.67	0.90	0.09	0.82
Depressive symptoms 1	Depressive symptoms	0.50	0.85	0.48	0.58	0.05	0.69
Depressive symptoms 2				0.55	0.63	0.04	0.74
Depressive symptoms 3				0.61	0.58	0.04	0.78
Depressive symptoms 4				0.35	0.56	0.06	0.59
Depressive symptoms 5				0.65	0.67	0.04	0.80
Depressive symptoms 6				0.35	0.51	0.06	0.59
Overall pain 1	Pain severity	0.62	0.86	0.80	1.48	0.10	0.90
Highest pain 2				0.63	1.21	0.12	0.79
Lowest pain 3				0.41	1.34	0.14	0.64
Current pain 4				0.62	1.59	0.12	0.79
Fibromyalgia impact 1	Fibromyalgia impact	0.67	0.81	0.64	2.32	0.17	0.80
Fibromyalgia impact 2				0.71	2.44	0.17	0.84

AVE = average variance extracted; CR = composite reliability; R^2^ = squared multiple correlation. B = unstandardized coefficient; *β* = standardized coefficient; SE = standard error. In the model, all β were significant at *p* < 0.001.

**Table 3:** Direct association estimates of the structural models.

M1					
Independent Variable		Criterion Variable	B	SE	*β*
Rumination	_	Physical fatigue	0.31	0.26	0.33
Rumination	_	Depressive symptoms	0.15	0.13	0.19
Rumination	_	Pain severity	0.13	0.31	0.08
Rumination	_	Fibromyalgia impact	0.60	0.59	0.19
Magnification	_	Physical fatigue	0.67	0.32	0.77 *
Magnification	_	Depressive symptoms	0.38	0.20	0.53
Magnification	_	Pain severity	0.18	0.40	0.12
Magnification	_	Fibromyalgia impact	0.25	0.83	0.09
Helplessness	_	Physical fatigue	0.76	0.24	0.73 **
Helplessness	_	Depressive symptoms	0.52	0.21	0.60 **
Helplessness	_	Pain severity	0.72	0.37	0.40 *
Helplessness	_	Fibromyalgia impact	0.19	0.79	0.07
Cognitive fusion	_	Physical fatigue	0.17	0.06	0.30 **
Cognitive fusion	_	Depressive symptoms	0.20	0.05	0.43 ***
Cognitive fusion	_	Pain severity	0.08	0.08	0.08
Cognitive fusion	_	Fibromyalgia impact	0.20	0.24	0.11
Physical fatigue	_	Depressive symptoms	0.06	0.07	0.08
Physical fatigue	_	Fibromyalgia impact	0.57	0.38	0.17
Depressive symptoms	_	Fibromyalgia impact	0.72	0.42	0.18
Pain severity	_	Physical fatigue	0.05	0.06	0.08
Pain severity	_	Depressive symptoms	0.04	0.03	0.08
Pain severity	_	Fibromyalgia impact	0.70	0.16	0.36 ***
**M2**					
**Independent Variable**		**Criterion Variable**	**B**	**SE**	***β***
Rumination	_	Fibromyalgia impact	0.78	0.23	0.25 **
Magnification	_	Physical fatigue	0.41	0.17	0.47 ***
Helplessness	_	Physical fatigue	0.77	0.21	0.73 ***
Helplessness	_	Depressive symptoms	0.21	0.08	0.24 **
Helplessness	_	Pain severity	0.71	0.17	0.40 ***
Cognitive fusion	_	Physical fatigue	0.17	0.07	0.26 **
Cognitive fusion	_	Depressive symptoms	0.19	0.05	0.35 ***
Physical fatigue	_	Depressive symptoms	0.16	0.06	0.19 *
Physical fatigue	_	Fibromyalgia impact	0.59	0.31	0.18 *
Depressive symptoms	_	Fibromyalgia impact	0.87	0.33	0.22 **
Pain severity	_	Fibromyalgia impact	0.69	0.15	0.36 ***

*** *p* < 0.001, ** *p* < 0.01, * *p* < 0.05. B = unstandardized coefficient; *β* = standardized coefficient; SE = standard error.
